# Household poverty in people with severe mental illness in rural China: 1994–2015

**DOI:** 10.1192/bjo.2020.95

**Published:** 2020-09-17

**Authors:** Yue-Hui Yu, Wei Luo, Man-Xi He, Xin Yang, Bo Liu, Yu Guo, Graham Thornicroft, Cecilia Lai Wan Chan, Mao-Sheng Ran

**Affiliations:** School of Public Administration and Policy, Renmin University of China, China; Xinjin Second People's Hospital, China; Chengdu Mental Health Center, China; Guangyuan Mental health Center, China; Jingzhou Mental Health Center, China; School of Labor and Human Resources, Renmin University of China, China; Centre for Global Mental Health, Institute of Psychiatry, Psychology and Neuroscience, King's College London, UK; Department of Social Work and Social Administration, The University of Hong Kong, Hong Kong SAR, China; Department of Social Work and Social Administration, The University of Hong Kong, Hong Kong SAR, China

**Keywords:** Severe mental illness, household poverty, social development, China

## Abstract

**Background:**

Little is known about poverty trends in people with severe mental illness (SMI) over a long time span, especially under conditions of fast socioeconomic development.

**Aims:**

This study aims to unravel changes in household poverty levels among people with SMI in a fast-changing rural community in China.

**Method:**

Two mental health surveys, using ICD-10, were conducted in the same six townships of Xinjin county, Chengdu, China. A total of 711 and 1042 people with SMI identified in 1994 and 2015, respectively, participated in the study. The Foster-Greer-Thorbecke poverty index was adopted to measure the changes in household poverty. These changes were decomposed into effects of growth and equity using a static decomposition method. Factors associated with household poverty in 1994 and 2015 were examined and compared by regression analyses.

**Results:**

The proportion of poor households, as measured by the headcount ratio, increased significantly from 29.8% in 1994 to 39.5% in 2015. Decomposition showed that poverty in households containing people with SMI had worsened because of a redistribution effect. Factors associated with household poverty had also changed during the study period. The patient's age, ability to work and family size were of paramount significance in 2015.

**Conclusions:**

This study shows that the levels of poverty faced by households containing people with SMI has become more pressing with China's fast socioeconomic development. It calls for further integration of mental health recovery and targeted antipoverty interventions for people with SMI as a development priority.

## Background

In the past few decades, China has experienced profound socioeconomic transformation that includes, but is not limited, to rapid economic growth, increased urbanisation and migration, transformed family structure and an enhanced social welfare system. Among these, economic growth is particularly striking, as gross domestic product has increased from 367.9 billion in 1978 to 90 030.9 billion in 2018. A positive outcome is that the population who live in extreme poverty have reduced from near 240 million in 1978 to 16.6 million in 2018. China has contributed over 70% of the global total achievement on poverty reduction.^1^ Despite this tremendous achievement, poverty alleviation is still an important issue for China, as new structured poverty traps have been created alongside the dramatic improvement. Poverty reduction for vulnerable groups such as people with mental illness has become even tougher.^2^ Instead of relying further on development, China has stepped into an era of targeted poverty alleviation.^3^

## Impact of severe mental illness on poverty

Compared with other vulnerable groups, people with severe mental illness (SMI) may be even more disadvantaged because of stigma and higher social stress.^4^ SMI is characterised as mental, behavioural or emotional disorders that can result in serious functional impairment. Commonly, SMI includes schizophrenia and mood disorders (e.g. major depressive disorder and bipolar disorder), which are leading causes of disability.^5,6^ Evidence strongly suggests that people with SMI are at increased risk of drifting into or remaining in poverty because of reduced productivity, increased medical costs, stigma and loss of employment-associated earnings.^7^ Meanwhile, a negative impact because of SMI also occurs at the household level, especially in societies that rely on the role of families in patient care.^8^ Having a family member with SMI is associated with treatment costs, caregiving and income loss.^9^ In general, those households are less likely to benefit from the trickle-down effect of external development.^5,10^

The most recent nationwide data from the China Mental Health Survey showed the weighted prevalence of any disorder (excluding dementia) in China was 16.6%, with the lifetime prevalence of schizophrenia and any mood disorder being 0.6 and 7.4%, respectively.^11^ Data regarding poverty among people with SMI is scarce in China. A survey suggests that there are 11.7 million adults with a mental disability, and around 38.2% of them are poor.^12^ Available data to show how many families have been affected by SMI and to what extent is rarer. What is known is that, according to the National Bureau of Statistics, the rate of social participation for people with SMI has declined in recent years to less than 50%. Given deep-rooted family collectivism,^13^ a broad impact of SMI on a household's economy may exist, and should be explored and addressed.

## Aims

Although household poverty among people with SMI has been documented in many studies,^9,14^ few of them has been conducted in China's development context or taken a dynamic perspective to view changes over time. Applying data from an ongoing mental health project in a less developed area of China, the objectives of this study were: (a) to examine the poverty trends of households containing people with SMI in the context of general socioeconomic development, and (b) to explore the reasons behind such changes. Consequently, this study compares different poverty measures for households containing people with SMI at two time points, and assesses the effects of growth and equity on those changes.^15^ Then, we test the relevant factors at each time points and compared their relative roles. This study may provide clues about household poverty in people with SMI during a period of development, thus providing evidence for further integration of mental health recovery and targeted poverty alleviation.

## Method

### Data source

This study was based on data from the Chengdu Mental Health Project (CMHP), an ongoing project on mental illness and mental health services in Xinjin county, Chengdu that started in the early 1990s. Data for this study were derived from two epidemiological mental health surveys in the same six townships of Xinjin county in 1994 and 2015.

In this study, SMI included schizophrenia and mood disorders. The ICD-10^16^ was applied as the diagnostic tool in both rounds of mental health surveys. The survey in 1994 covered a population of 123 572 (≥15 years old) and diagnosed 711 people with SMI (schizophrenia: *n* = 515; mood disorders: *n* = 196). Another round of the survey in 2015 covered a population of 152 776 (≥15 years old) and revealed a total of 1042 people with SMI (schizophrenia: *n* = 671; mood disorders: *n* = 371).

Detailed methods regarding the two surveys have been described elsewhere.^5,16,17^ Briefly, both surveys were completed in two steps. First, the Psychoses Screening Schedule^5^ was completed in face-to-face interviews with household heads to identify potential individuals with mental disorders. Key information from village doctors and neighbours was also considered. Second, trained psychiatrists conducted a comprehensive general psychiatric interview with the person with a potential mental disorder for further diagnosis. The instruments employed in the survey in 2015 were modified based on those used in 1994 and some new variables such as social welfare, stigma and social support were added. The measurement of most variables selected for use in the present study were the same in 1994 and 2015. The ICD-10 was applied as the diagnostic tool in both rounds of surveys.

These surveys were approved by the University Human Research Ethics Committee. All research participants provided informed consent after receiving a complete description of the study.

Chengdu is a provincial city in Western China. Compared with first-tier cities, it is less developed. Nevertheless, the surveyed county has also experienced huge development during the past few decades. In 1994, when the CMHP group conducted the first survey, Xinjin county was a representative middle-income rural county in Southwestern China. It had shifted to being one of the most favoured places for investment in Western China when the CMHP group conducted the second survey in 2015. According to the data from local National Bureau of Statistics, the residential income in Xinjin has experienced substantial growth, with per capita disposable income increased from 4014 Chinese yuan (CNY) (urban residents) and 1757 CNY (rural residents) in 1994 to 31 637 CNY (urban residents) and 18 492 CNY (rural residents) in 2015. The development of Xinjin is an epitome of China, thus it is representative to achieve the abovementioned research objectives.

### Analytic strategy

Poverty in this study refers to monetary poverty. Households with a reported annual income per capita lower than the poverty standard were defined as poor. The minimum living standard in Xinjin, defined by the Chengdu government, was applied as the poverty standard; these were 850 CNY and 5400 CNY, respectively, for the years 1994 and 2015. The standard in 1994 was adjusted based on that of the 2015 Consumer Price Index (CPI). Analysis in this study includes: (a) comparison and decomposition of poverty; and (b) regression analysis.

First, household income and poverty at two time points were compared. The well-known Foster-Greer-Thorbecke (FGT) poverty index^18^ was applied, in which poverty was broken down into three aspects: breadth (P_0_); depth (P_1_) and severity (P_2_). P_0_ describes the headcount ratio of poor households, P_1_ describes the gaps between poverty status of poor households and the poverty standards and P_2_ describes the status of the poorest poor among the group. The FGT index synthesised the idea of poverty breadth, depth and severity in one equation as illustrated in equation (1), in which x_i_ represents the per capita income of a household i, *n* is the total number of households, z is the poverty line and α (≥ 0) is the degree of aversion to inequality.
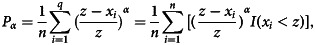
whereequation 1
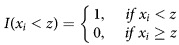


All poverty measures of P_0_, P_1_ and P_2_ in 1994 and 2015 were compared, and the changes were further decomposed using a widely applied static decomposition method.^19,20^ In this decomposition, a poverty measure *P_t_* at date *t* is characterised in terms of the poverty line (*z*), the mean income of the distribution (*u_t_*) and the Lorenz curve (*L_t_*), which represents the relative income inequalities. *P_t_* is written as equation 2:equation 2
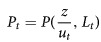


Correspondingly, the growth component of change is defined as the change in poverty because of a change in mean income while holding the Lorenz curve constant at some reference level *L_r_*. The redistribution component means a change in poverty because of the change in the Lorenz curve while the mean income remains constant at the reference level *u_r_*, thus the growth effect and redistribution effect on changes in poverty can be decomposed as equation 3:equation 3
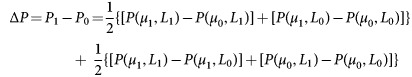


After this, both linear and logistic regressions were performed to estimate the roles of relevant factors. For the purpose of comparison, the same variables in 1994 and 2015 were selected. Variables selected for regressions were based on literature review and data availability. Evidence showed that variables that might influence the income and poverty status of households included ability to work, physical health status, disability level, household size, social support and caring burden for people with SMI.^21–25^ Household size was defined as the number of people who ‘eat from the same pot or live in the same residential unit’, as reported by the respondents.^8^

Given the different socioeconomic development level of each township, household location might also differentiate household poverty among people with SMI.^26^ Based on available data-sets, four blocks of variables were put in the regressions analysis, which included (a) patient's sociodemographic characteristics (i.e. age, gender, marital status and education); (b) competence-related indicators (i.e. diagnosis of SMI, duration of mental illness, ability to work, physical illness and disability level), (c) the household-level factor (i.e. household size) and (d) household location (i.e. the townships in which these households resided).

## Results

### Sociodemographic characteristics

Table 1 shows participant's sociodemographic and clinical characteristics at different time points (1994 and 2015). In 1994, there were 515 (72.4%) people with schizophrenia and 196 (27.6%) people with mood disorders. In 2015, there were 671 (64.4%) people with schizophrenia and 371 (35.6%) people with mood disorders. More people with mood disorders were included in this study in 2015 than 1994 (*P* < 0.001).

**Table 1:** Sociodemographic and clinical characteristics of people with severe mental illness in 1994 and 2015

	1994 (*n* = 711)	2015 (*n* = 1042)	*P*
Gender, male: *n* (%)	338 (47.5)	471 (45.2)	0.360
Occupation, peasant: *n* (%)	706 (99.3)	930 (89.3)	<0.001
Work ability, unable to work: *n* (%)	106 (14.9)	195 (18.7)	0.044
Physical illness, yes: *n* (%)	33 (4.6)	373 (35.8)	<0.001
Marital status, married: *n* (%)	471 (66.2)	651 (62.5)	0.118
Educational level, *n* (%)			<0.001
≥High school	31 (4.4)	235 (22.6)	
Primary and middle school	551 (77.5)	639 (61.3)	
Illiterate and unknown	129 (18.1)	168 (16.1)	
Diagnosis, *n* (%)			<0.001
Schizophrenia	515 (72.4)	671 (64.4)	
Mood disorders	196 (27.6)	371 (35.6)	
Social function (mental disability level), *n* (%)			<0.001
Extremely serious	190 (26.7)	221 (21.2)	
Serious or moderate	79 (11.1)	176 (16.9)	
Mild or non-disabled	442 (62.2)	645 (61.9)	
Age, years: mean (s.d.)	44.5 (15.6)	56.0 (14.2)	<0.001
Duration of mental illness, years: mean (s.d.)	12.3 (11.7)	17.3 (13.8)	<0.001
Household size, mean (s.d.)	3.4 (1.5)	3.0 (1.5)	<0.001
Annual income per person (CNY),^a^ mean (s.d.)	1110 (923.3)	8420 (7927.1)	<0.001
Income Adjusted by CPI, 1994 as base:^b^ mean (s.d.)	1110 (923.3)	4651 (4379.5)	0.006

CNY, Chinese yuan; CPI, Consumer Price Index.

a. The per capita disposable income increased from 4014 CNY and 1757 CNY in 1994 to 31 637 CNY and 18 492 CNY in 2015 for urban and rural residents, respectively, in Xinjin county.

b. Adjusted by CPI, the income for urban and rural residents in 2015 were 17 479 CNY and 10 216 CNY, respectively.

There were substantial changes in the characteristics of people with SMI and their households in Xinjin county. Compared with households containing people with SMI in 1994, participants in 2015 had a significant lower proportion of people who were peasants (*P*<0.001), although the proportion of peasants was still very high (89.3%). There was also a higher proportion of people who were unable to work (18.7%, *P* < 0.05), with physical illness (35.8%, *P* < 0.001), who had attained a higher level of education (22.6%, *P* < 0.001), as well as a lower rate of people with extremely serious mental disability (21.2%, *P* < 0.001). In addition, participants in 2015 were significantly older and had a longer duration of mental illness than those in 1994 (*P* < 0.001).

The household size in 2015 was significantly smaller than that in 1994 (*P* < 0.001). The annual household income per person had increased significantly from 1110 CNY in 1994 to 8420 CNY in 2015 (*P* < 0.001). Adjusted for the CPI, the annual household income per person in 2015 were 4651 CNY, which was significantly higher than that in 1994 (*P* < 0.01). The annual disposable income for urban and rural residents in Xinjin in 1994 and 2015 are reported as a footnote in Table 1. For both years, the annual income for households with people with SMI was lower than that of the rural average in Xinjin.

### Household income and inequality in 1994 and 2015

**Fig. 1:**
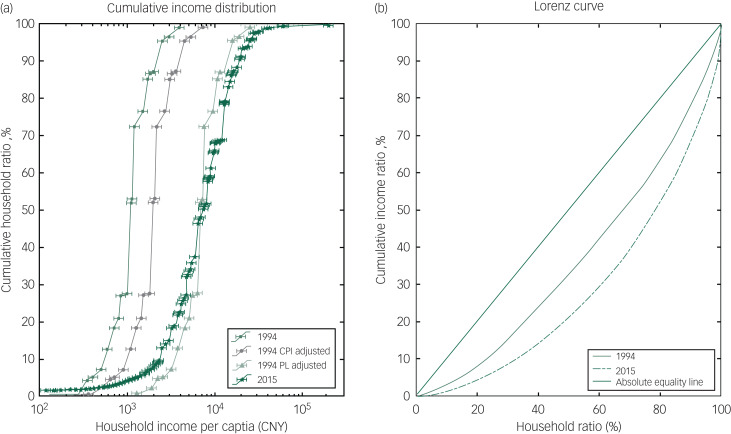
Household income distribution of people with severe mental illness in 1994 and 2015.

CNY, Chinese yuan; CPI, Consumer Price Index; PL, poverty line.

Figure 1 illustrates the income distributions among households containing people with SMI in 1994 and 2015. The income distribution curve in 2015 appears on the far right in Fig. 1(a), showing that the household income per capita in 2015 was much higher than that in 1994 and also after adjustment for CPI. However, the distribution of wealth in 1994 adjusted by the poverty standard in Fig. 1(a) crossed with that in 2015 at the cumulative household ratio of around 50%, which means the economic status of near 50% of households were worse than their counterparts when the poverty standard was considered. In other words, the income inequality in households containing people with SMI has increased in 2015. Figure 1(b) further demonstrates the income inequality with the Lorenz curve. Compared with 1994, the distribution of cumulative income in 2015 deviated further from the diagonal line.

### Changes in household poverty and its decomposition

Table 2 shows the changes in household poverty for different measures. The proportion of poor households, as measured by the headcount ratio (P_0_), increased significantly from 29.8% in 1994 to 39.5% in 2015 (*P* < 0.001). A significant rise in P_1_ (*P* < 0.05) from 7.5 to 11.8 indicated the average income of poor households had decreased. Although P_2_ – the indicator of poverty severity – had also increased from 3.8 to 6.8, the test of difference was not significant (*P* = 0.102).

**Table 2:** Changes in poverty (P) measures and the decomposition of people with SMI, 1994–2015

FGT index, diagnosis	1994^a^	2015^a^	Total effect^b^	*P*	Growth effect^c^	Redistribution effect^c^
P_0_ (poverty breadth)	29.8	39.5	9.7	<0.001	−44.8	54.5
Schizophrenia	30.7	40.7	10.0	0.006	−42.2	52.2
Mood disorders	27.6	37.2	9.6	0.002	−43.6	53.2
P_1_ (poverty depth)	7.5	11.8	4.3	0.015	−36.3	40.5
Schizophrenia	10.7	16.0	5.3	0.042	−35.1	40.4
Mood disorders	6.5	8.1	1.6	0.079	−35.5	37.1
P_2_ (poverty severity)	3.8	6.8	3.0	0.102	−29.2	32.2
Schizophrenia	5.8	8.1	2.3	0.093	−29.2	31.5
Mood disorders	2.8	5.1	2.3	0.081	−28.6	30.9

FGT, Foster-Greer-Thorbecke.

a. All calculated values have been multiplied by 100.

b. Total effect equal to the rise of P _(*n* = 0, 1, 2)_ in 2015, compared with that of 1994. The *P*-value is the significance test of each poverty measure in 1994 and 2015. The test was adjusted by the degree of freedom (d.f.), sampling weight and household size.

c. In the decomposition, positive values indicate poverty growth whereas negative ones indicate poverty alleviation.

In terms of household poverty by diagnosis of SMI, in general, poverty faced by households of people with schizophrenia was worse at both time points, as all numbers for P_0_, P_1_ and P_2_ were larger than those for people with mood disorders. Meanwhile, the increase in P_0_ and P_1_ were also larger for people with schizophrenia.

Growth and redistribution had contributed differently for the abovementioned changes in poverty, as all numbers on growth effect were negative while they were positive in the column for redistribution effect (Table 2). Increased income inequality (i.e. redistribution effect) was the main reason behind the deterioration in poverty levels from 1994 to 2015 for those households containing people with SMI. For example, an 11.9% increase in P_0_ could be explained by a pure growth effect of −42.6% and a pure redistribution effect of 54.5%. In other words, if growth had not changed, the redistribution in 2015 would have increased the household poverty ratio by 54.5%. Compared with the redistribution effect, values on growth effect were negative but smaller.

### Factors associated with household poverty in 1994 and 2015

Table 3 shows the results of regression analysis for household income and poverty status. Patient's educational attainment, diagnosis of SMI, duration of mental illness and household location in different townships were not significantly associated with household poverty in both 1994 and 2015 (*P*>0.05). Instead, age and the household size had a salient relationship with household poverty at both time points (*P* < 0.05). Specifically, younger patients and a larger family size had a significant positive relationship with higher household income and being above the poverty line. Households with patients who were male, unmarried and severe mentally disabled were worse off. Patient's ability to work was a factor that was only significant in 2015, as being unable to work was associated negatively with household income and status of above the poverty line (*P* < 0.001).

**Table 3:** Associative factors for household income and poverty status in 1994 and 2015

	Linear regression models (DV: income)				Logistic regression models (DV: poor = 1, non-poor = 0)			
	1994		2015		1994		2015	
	*B* (95% CI)	*P*	*B* (95% CI)	*P*	*B* (s.e.)	*P*	*B* (s.e.)	*P*
Constant	3.251 (3.045 to 3.457)	0.021	3.247 (2.779 to 3.715)	0.040	−1.482 (0.276)	0.032	−1.099 (0.231)	0.031
Age	−0.011 (−0.017 to −0.005)	0.013	−0.024 (−0.048 to 0.000)	0.003	0.013 (0.010)	0.011	0.023 (0.009)	<0.001
Female (male = 0)	0.039 (−0.030 to 0.108)	0.025	0.142 (−0.072 to 0.356)	0.062	−0.243 (0.184)	0.026	−0.195 (0.132)	0.074
Unmarried (married = 0)	−0.102 (−0.184 to −0.020)	0.032	−0.091 (−0.471 to 0.289)	0.194	0.482 (0.139)	0.007	0.109 (0.123)	0.082
Education (illiteracy = 0)								
≥High school	−0.163 (−0.365 to 0.039)	0.132	0.028 (−0.319 to 0.375)	0.458	0.520 (0.484)	0.193	−0.137 (0.234)	0.875
Primary and middle school	0.018 (0.006 to 0.030)	0.214	0.079 (−0.115 to 0.273)	0.928	−0.019 (0.252)	0.172	−0.142 (0.187)	0.650
Schizophrenia (mood disorders = 0)	0.002 (−0.098 to 0.102)	0.704	0.291 (0.048 to 0.534)	0.562	−0.253 (0.209)	0.602	−0.057 (0.142)	0.408
Duration of mental illness	−0.006 (−0.008 to −0.004)	0.988	−0.004 (−0.014 to 0.006)	0.074	−0.006 (0.004)	0.844	0.002 (0.003)	0.521
Unable to work (able to work = 0)	−0.075 (−0.177 to 0.027)	0.146	−0.727 (−0.980 to −0.474)	0.000	0.402 (0.103)	0.091	0.524 (0.148)	<0.001
Physical illness (no = 0)	0.019 (−0.216 to 0.254)	0.112	−0.062 (−0.340 to 0.216)	0.061	−0.411 (0.253)	0.062	0.240 (0.134)	0.061
Disability level (mild or non−disabled = 0)								
Extremely serious	−0.188 (−0.270 to −0.106)	0.004	−0.205 (−0.640 to 0.230)	0.696	0.672 (0.242)	0.005	0.198 (0.104)	0.255
Serious or moderate	−0.069 (−0.177 to 0.039)	0.862	0.072 (−0.228 to 0.372)	0.301	0.553 (0.389)	0.241	−0.020 (0.091)	0.162
Household size	0.038 (−0.011 to 0.087)	0.008	0.096 (0.039 to 0.153)	0.040	−0.111 (0.039)	0.041	−0.108 (0.052)	0.004
Location (Huayuan township = 0)								
Xinyi	−0.402 (−0.504 to −0.300)	0.273	−0.525 (−1.005 to −0.045)	0.129	−0.429 (0.120)	0.082	−0.524 (0.424)	0.152
Xinping	0.074 (−0.008 to 0.156)	0.174	0.472 (0.252 to 0.692)	0.082	0.038 (0.251)	0.124	0.452 (0.092)	0.097
Huaqiao	0.624 (0.116 to 1.132)	0.067	0.735 (0.241 to 1.229)	0.058	0.553 (0.224)	0.079	0.452 (0.024)	0.371
Puxing and Anxi (in 2015)	−0.245 (−0.408 to −0.082)	0.249	−0.320 (−0.330 to −0.310)	0.235	−0.057 (0.195)	0.263	−0.302 (0.012)	0.229
Fangxing and Wenjing (in 2015)	−0.525 (−0.582 to −0.468)	0.092	−0.625 (−0.676 to −0.574)	0.098	−0.245 (0.139)	0.129	−0.205 (0.113)	0.082
Summary statistics								
Adjusted *R*_2_	0.58		0.71					
Chi-squared test					89.035		119.466	
−2 Log likelihood					908.592		132.593	
Pseudo *R*_2_					0.183		0.259	

DV, dependent variable; non-poor, above the poverty line.

Table 4 illustrates the standardised coefficient (i.e. beta) and the odds ratio (i.e. Exp (*B*)) for the significant associative factors with household poverty that are detailed in Table 3. In 1994, having a person with severe mental disability in a household was the most important factor explaining household poverty (*B* = −0.219 and Exp (*B*) = 2.008 for the status of being severe mentally disabled), followed by a patient's marital status, gender, age and the household size. The most important factor that was associated with household poverty in 2015 was patient's ability to work (*B* = −0.306 and Exp (*B*) = 1.129 for the status of being unable to work), followed by a patient's age and the household size. Household size was the least significant factor at both time points.

**Table 4:** Standardised coefficient and odds ratio of significant factors in 1994 and 2015

	Household size	Age	Female	Unmarried	Unable to work	Severe mental disability
1994						
Beta	0.109	−0.115	0.131	−0.169	—	−0.219
Exp (B)	0.830	1.102	0.693	1.635	—	2.008
2015						
Beta	0.068	−0.112	—	—	−0.306	—
Exp (B)	0.798	1.093	—	—	1.129	—

## Discussion

### Main findings

This study provides a profile of changes in poverty in households containing people with SMI in the context of China's rapid socioeconomic development. To the best of our knowledge, this is the first attempt to study changes in household poverty in people with SMI over a long time span (1994–2015). Our findings suggested that although mean income was significantly higher in 2015 for households of people with SMI (*P* < 0.01), wealth distribution had also become more unequal, as the Lorenz curve in 2015 deviated further from the line that represented equal wealth distribution. This study adds to evidence demonstrating increased income inequality along with general raised mean income in China.^27^ Not only did the income gap between the households of people with SMI and other households increase,^17^ but also the income inequality within this unique group has increased.

The results of this study show that the situation regarding levels of poverty faced by households containing people with SMI in 2015 was even worse than that faced by their counterparts in 1994; all measures on poverty breadth, depth and severity were higher. This is inconsistent with China's general context of poverty reduction.^28^ Further static decomposition suggested that the anomaly was rooted in increased income inequality, as all changes in poverty measures were dominated by the redistribution effect. The growth effect had contributed negatively to poverty deterioration but it was insufficient to off-set the role of income inequality. It indicates that poverty among people with SMI is not likely to be alleviated automatically through promoting fast growth. Instead, increased inequality alongside economic growth has further aggravated poverty in the households contain people with SMI. These findings are consistent with earlier views of an association between poverty and inequality.^15^ To achieve the goal of reducing poverty requires strong, country-specific combinations of both growth and distribution policies.^29^

### Eradicating poverty

Although the poverty trends for households containing people with SMI were not optimistic, the results of this study indicated that poverty severity (P_2_) had not increased significantly from 1994 to 2015. In other words, the poorest poor among people with SMI had not fallen further below the poverty line. This may be partly explained by basic social welfare supplies for such households, include the basic living allowance, subsidy for disabled people and endowment insurance that matches with disability assessment.^30^

Further targeted poverty alleviation programmes are still needed to reduce poverty, especially its breadth and depth. Poverty measures in this study are also helpful in estimating the size of resources needed to ‘eradicate’ poverty.^31^ If policy implementation was possible to perfectly allocate resources to the poor, then, in 2015 a total amount of 0.65 million CNY (P_1_ ×  the poverty line × surveyed population) would have been needed to raise the income of all poor households of people with SMI above the poverty line in Xinjin county.

### Relevance of dependency ratio

Apart from knowing how socioeconomic development had shaped the trends in household poverty, regression analyses were employed to reveal possible factors that related to household poverty at different times among these unique households. Larger household size and younger age of people with SMI had salient positive relationship with better income and a non-poor status of a household in both the years of 1994 and 2015. This may be explained by the dependency ratio of a household that contains people with SMI. SMI can restrict a person's labour productivity and, in this case, larger household size may lower the dependency ratio of those households thus maintaining a relative higher income.^24^ Similarly, patients with SMI of younger age may have not become the main income earner of a household thus their influence on household poverty may be relatively smaller.

For older people with SMI, the caregiving burden for patients may overlap with elderly care, a situation in which caregiving may result in a higher dependency ratio. What really matters for household poverty may be the dependency ratio rather than the household size or the age of the patient.^32^

### Gender, marital status and severity of illness

The results of this study showed that being male, being unmarried and severe mental disability were positively associated with household poverty in 1994. The reason for gender differences may be that a labour force of men is more important in a rural agricultural areas,^33^ which was the context of Xinjin in 1994, thus the negative impact associated with men with SMI was more severe. The association between patient's status of being unmarried and household poverty can be partly explained by social support that linked tightly with marriage in Chinese rural culture. Without marriage, the social network for reciprocating favours and income earning could have been restricted.^34^ Patients from low-income families might be disadvantaged in marriage.^35^ This may also be one reason that explains the association between marriage and household poverty.

After 21 years of development, Xinjin county had been transformed from a rural agriculture-dominated county into one of the most favoured places for investment in Western China. The role of gender and marital status of people with SMI were insignificant in the new context of 2015. Because of basic social welfare, the association between mental disability and household poverty, which was the strongest association in 1994, was also insignificant in 2015. The new additional significant factor in 2015 was a patient's ability to work. Again, this can be explained by the dependency ratio of a household, as being unable to work means a higher dependency ratio. In the previous rural context (i.e. 1994), work ability cannot differentiate household income because the labour supply in rural areas was more likely to be redundant, meaning that many people may not have been employed.^36^ Whether a person with SMI can work or not in a rural context may not be as important as in the current context of urbanisation. In the current context, although the majority of people with SMI were still working in farming, those with the ability to work may search for extra part-time work opportunities.

### Social development theory and SMI

Although China has achieved remarkable economic growth and an impressive record of poverty reduction over the past four decades, poverty, especially relative poverty, still remains a key challenge.^5,37^ The results of this study also support the social development theory of people with mental disorders,^5^ that is, social development has a strong impact on the pace and direction of poverty changes for people with mental disorders. Currently, China has the national strategic aim of targeted poverty alleviation.^3^ However, no specific, targeted antipoverty policies have been formulated for households containing people with SMI. This study provides solid evidence that shows how overall social prosperity has contributed to poverty deterioration for those households containing people with SMI. Formulating policies to alleviate poverty in households with people who have SMI and improving mental health recovery for people with SMI should be a priority for China in the context of the millennium development goals.^38^

### Implications

There are two major policy implications of this study. First, given that the trickle-down effect of economic growth, which highlights overall economic growth or increasing, is ineffective in tackling poverty resulting from income inequality, further redistribution policies should be formulated to narrow income gaps. At the very least, poverty severity (P_2_) should be further lowered and eradicated through targeted social welfare programmes. In addition, lowering poverty breadth (P_0_) and depth (P_1_) should also be considered.

Second, the impact of SMI on household economic status, as has been illustrated in this study, requires further antipoverty policies to be formulated for both people with SMI and their families. On the one hand, policymakers should seriously consider how to strengthen the current mental health system, especially community-based mental health services for people with SMI, which are essential for them to improve work and other social functions.^39^ On the other hand, the integration of mental health recovery and precise poverty alleviation programmes and culture-specific family interventions to empower the whole family should also be a focus.^16,17^ For example, taking the dependency ratio of a household into consideration and formulating differentiated antipoverty strategies.

### Limitations

The limitations of this study include the following. First, this study is rooted in the Chinese context of rapid socioeconomic development in the recent quarter of a century, and is based on a strong culture of family collectivism, therefore the results of this study may not be fully applicable to other upper-middle-income countries or those societies with strong individualism and weak family care.

Second, studying trends with only two time points separated by 20 years is also a limitation, as what has happened in between these time points is not included. Considerable variations may also exist due to differences in the measures used between the two rounds of surveys, which can be a problem for studies that analyse changes.^39^ Third, regression analysis in this study was applied to two waves of cross-sectional data; a causation relationship cannot be verified using this approach. Further studies may benefit from adopting a longitudinal design to examine the exact role of SMI on poverty and changes over time.

Fourth, because of limitations of the data some important variables for analysing household poverty such as family structure and dependency ratios^40^ were not included. Future studies should include more household-level factors as control variables.

Finally, only data for people with SMI were available for this research. Therefore, this research cannot reveal the level of poverty faced by households that did not contain people with SMI, or the general population. Further studies should be conducted to explore household poverty that includes both households with and without people with SMI.

In conclusion, this study has generated evidence that shows how conditions of poverty can worsen in the context of social prosperity. Household poverty, especially relative poverty, among people with SMI is not likely to be alleviated automatically within the process of socioeconomic development. Instead, growth has resulted in an increased income inequality and further aggravated poverty for households containing people with SMI. Our conclusions support the need for more intense and targeted antipoverty policies and programmes for households containing people with SMI. Targeted antipoverty policies and programmes should be developed for all people including those with SMI who are in absolute and relative poverty, and these policies and programmes are crucial for achieving the strategic goal of Healthy China 2030.^17^

## Data Availability

The data are not publicly available because they contain information that could compromise the privacy of research participants.
